# Influential Factors for Sustainable Intention to Visit a National Park during COVID-19: The Extended Theory of Planned Behavior with Perception of Risk and Coping Behavior

**DOI:** 10.3390/ijerph182412968

**Published:** 2021-12-08

**Authors:** Bo-Hyun Seong, Youngseok Choi, Hyojin Kim

**Affiliations:** 1 Department of Co-Prosperity Research, Chungbuk Research Institute, Cheongju 28517, Korea; sbh@cri.re.kr; 2 Department of Strategic Planning, Incheon Tourism Organization, Gaetbeol-ro 12, Yeonsu-gu, Incheon 21999, Korea; creatourism@naver.com; 3 Department of Tourism Management, Mokpo National University, Yeongsan-ro 1666, Cheonggye-myeon, Muan-gun 58554, Korea

**Keywords:** COVID-19, perception of risk, coping behavior, extended theory of planned behavior, sustainable intention

## Abstract

Despite the danger of the spread of the COVID-19 pandemic, visits to natural tourism destinations such as national parks are continuing, though people are using less congested trails or minimizing personal contact. Given the danger from COVID-19, the purpose of our study was to use an expanded theory of planned behavior to analyze whether tourists intend to continue to visit national parks. Another purpose for our study was to compare an extant research model based on the theory of planned behavior with the extended model we developed. Frequency analysis, confirmatory factor analysis, structural equation modeling, and other statistical techniques, such as correlation analysis, parsimonious fit index, and squared multiple correlations were employed according to the appropriate objectives. Additionally, the number of 351 participants joined the survey. Our study found that perception of risk of COVID-19 negatively affected attitude and perceived behavioral control in both models. Moreover, the perceived behavioral control had a positive effect on coping behavior. Given the analytical results, our study presents not only theoretical implications for understanding the behavior of those who visit national parks, but also practical implications for operation and management of national parks during the COVID-19 pandemic.

## 1. Introduction

The global shock from the COVID-19 pandemic has continued. In the past, people have generally recovered from either large or small infectious disease pandemics within about five months, but there is a difference between the past and the present, in that it is difficult to guarantee a complete end to COVID-19 during 2021 [1,2]. For tourism, this uncertainty is having an unprecedented impact on both the inbound and the outbound tourism markets [3,4]. Although the international tourism market continues to stagnate, it is noteworthy that the domestic tourism demand is robustly recovering in several countries [5]. People’s desire for tourism can quickly overcome considerable difficulties because of resilience, which is the strength of the mind after a major crisis, even if it is under unexpected cases, such as a pandemic or terrorism [6,7]. Indeed, in Korea there has been a clear increase in visitors around cities among environment-friendly destinations, such as national parks, where movement is easy and safe, depending on the intensity of COVID-19 countermeasures. The social concern driven by the pandemic can emerge as avoidance of travel, but at present our study is still significant, because natural or environmental destinations are being visited consistently and steadily [8,9].

In addition, if advice from experts that the cycle of the pandemic can be shortened is considered, participation in outdoor recreation to meet the desire for travel is likely to continue. Additionally, for effective management of areas for outdoor recreation, such as national parks, a fundamental discussion of the psychological factors leading to visiting parks is required. The theory of planned behavior is among the most frequently used theories in relation to intentions to visit national parks [10,11,12]. Plus, the usefulness of a theory of planned behavior as a theoretical framework for explaining various human behaviors, including tourism, is well-known in academia [13,14,15,16,17]. Constructs of the theory of planned behavior that involve attitude, subjective norms, and perceived behavioral intentions have already identified their significant relations to behavioral intentions by tourists in diverse research [18,19,20,21]. According to Ajzen (1991) [22], attitude is defined to be a positive or negative evaluation of specific conduct determined by an individual. Subjective norms indicate positive or negative opinions about specific conduct that a particular group around an individual exhibits. Perceived behavioral control accounts for the perceived level of ease or difficultly a specific action presents to an individual [23]. As an additional example to investigate human behavior, a study on whether constructs of the theory of planned behavior significantly influence behavioral intention in the setting of a national park is essential. In recent years, a wide array of studies have attempted to expand the theory of planned behavior by adding new factors to the variables in the theory, such as attitude, subjective norms, and perceived behavioral control, and to increase its power to explain tourist behavior, thereby contributing to better understanding of the additional influential factors [23]. New study on applications of the extended theory of planned behavior should attempt to explain the complicated behavior of humans in relation to the unpredicted COVID-19 pandemic [13,16,17,24,25].

Due to the outbreak of the COVID-19 pandemic, it is questionable whether tourists visit national parks that are at risk as they would otherwise plan to. Both this question and the rest of our study can be related to protection motivation theory, which deals with cognitive responses such as threat appraisal and coping approval derived from fear appeals [26,27,28]. Apart from protection motivation theory, our study used the expanded theory of planned behavior to inquire whether tourists intend to continue to visit a national park if they minimize their risk by means of countermeasures, such as using less crowded national trails and having less personal contact. Our study used data about visits to natural destinations such as national parks under the perception of the danger from COVID-19. Extant studies have found that the higher the risk perceived by tourists, the more they switch to other destinations or reduce their risk at the given tourism destination [23,29]. Our finding suggests other variables that involve perception of risk and coping behavior regarding COVID-19 that can be added to extend the theory of planned behavior [30,31]. As an additional purpose for our study, we compared the simple research model using the theory of planned behavior with the extended research model of our study. By comparing the two research models, our study identified ways to improve their explanatory power. To achieve these objectives, we established research hypotheses based on literature pertaining to tourist behavior, such as an extended theory of planning behavior, perception of risk, and coping behavior. Given the results from the quantitative analyses, our paper presents theoretical implications for the field of tourist behavior that are of practical importance for the management of environment-friendly tourism destinations, such as national parks, and presents future research that is needed because of the limitations of this research.

## 2. Literature Review

### 2.1. COVID-19 and Perception of Risk

Risk is perceived if there is a possibility that the goods and services provided may be harmful or have undesirable consequences [32]. People intuitively feel danger, which is called perception of risk [33]. The previous literature proposes that a risk is perceived not objectively and probabilistically, but subjectively in a specific situation [34]. For tourism, perception of risk is described as the anxiety that tourists experience while purchasing and consuming tourism products and services [35]. Likewise, the definition of the perception of risk from COVID-19 from the research model of our study is the subjective anxiety of an individual when he or she visits national parks during the pandemic. When tourists perceive a high risk while searching for information before visiting a tourism destination, they are influenced by their cares and worries, thereby influencing their satisfaction with and loyalty to tourism products and destinations [36,37,38].

In the previous literature, the factors that caused tourists to perceive danger were classified into seven categories: health, political instability, terrorism, unfamiliar food, cultural barriers, national political or religious doctrine, and misdemeanors [39]. Likewise, Dolnicar (2005) [40] claimed that risks consist of political risks, such as terrorism, political instability, and military conflicts; environmental risks, such as natural disasters and landslides, and difficulties with accessing hospitals; physical risks, such as life-threatening diseases and possible unmet needs of clean food and water; risks in planning because of unreliable aviation and inexperienced operators; and financial risks, such as theft and loss of luggage. The biggest constraint on tourist behavior in 2020 was the spread of COVID-19, which is expected to continue throughout 2021 at least. Infectious diseases—included by many studies on risk perception in the tourism sector [39,40,41]—are assumed to be a major constraint on tourist behavior. As a recent example, compared to the period of January to June 2019, the number of visitors to major tourism spots in Korea in 2020 decreased by 40–60% in various regions, which shows an unprecedented shock to the whole tourism industry [42].

On the other hand, the decrease in visitors to national parks differs from the decreases in other at tourism destinations. According to the Korea National Park Service (2021) [43], the number of visitors to national parks in 2020 decreased by 19.2%, from 19,899,596 to 16,081,996, compared with January to June 2019, which needs attention by researchers, because that is a relatively small decrease compared to the decreases in tourism to other destinations. This was because travel was suppressed in the aftermath of COVID-19 and was replaced by outdoor recreation, which is judged to be comparatively safe. People are coping by means of strategies for risk reduction: tourists tend to avoid purchasing tourism products when the risk perceived by them exceeds a permissible level [30,44,45,46].

### 2.2. Extended Theory of Planned Behavior

The theory of planned behavior (TPB) by Ajzen (1991) [22] was proposed by extending the Fishbein and Ajzen’s (1975) [47] theory of reasoned action (TRA) [24]. It has often been referred to in studies on the decision-making process in which individuals displayed specific behaviors by means of a few variables, such as attitude, subjective norms, and perceived behavioral control [42,48]. As addressed earlier, each of the constructs from the theory of planned behavior is briefly explained as follows. The definition of attitude is a positive or negative assessment of specific conduct determined by an individual. Subjective norms are defined to be positive or negative opinions about specific conduct that a group has in a society. Perceived behavioral control is defined to be the perceived level of ease or difficulty an individual feels in regard to specific conduct [22]. The theory of planned behavior is the dominant theory that accounts for human behavior in many fields, such as health, learning, consumer, environment friendliness, and tourism [18,19,25,49,50,51,52,53]. Even though this is not always the case, the low explanatory power of three independent variables including attitude, subjective norms, and perceived behavioral control, for behavioral intentions [54,55,56,57], has sometimes led to other independent variables being added to extend the theory and strengthen its explanatory power for human behavior [58]. Even when many additional predictors are added to the theory though, there is the possibility of providing no improvement to the theory. Nevertheless, we created an extended theory of planned behavior which includes perception of risk from COVID-19, plus coping behavior and sustainable intention to visit. We then proved its validity by finding that is has more explanatory power than the original theory of planned behavior [20,23,59]. Although when a new independent or mediating variable is added to the original theory, it is a radical change—for example, how the model of goal directed behavior is distinguished from the theory of planned behavior—we also attempted to extend and investigate the theory [60].

Previous literature has increased the understanding of tourist behavior and has encouraged the development of theories that apply micro-psychological factors, such as past experience, prior knowledge, motivation, imagination, pleasure, and involvement; and external environmental factors, such as risk perception, structural constraints, and social class, to the expansion of the theory of planned behavior [21,61,62,63,64,65,66,67,68]. A few previous studies that have expanded the theory of planned behavior by using risk perception have continued to verify the significance of risk perception as an antecedent variable that affects major variables from the theory [67]. As examples of representative studies, Yoon et al. (2010) [69] argued for the significance of the relationship between perceived risk and attitude in overseas travel, and Quintal et al. (2010) [67] claimed that risk perception by outbound travelers from Korea, Japan, and China had a significant effect on their attitudes. Additionally, Lee and Kim (2017) [70] maintained that the perception of risk from fine dust had a significant effect on attitudes and subjective norms toward outdoor recreation activities. Both studies on the positive relationship between risk perception and risk-reducing behavior, and research on coping behavior led by psychological variables derived from attitudes formed by experiencing risks, imply that a study on the expansion of the theory of planned behavior with variables of risk perception and coping behavior about COVID-19 would be significant [31,34,71,72,73,74].

### 2.3. Coping Behavior

Manning (1986) [75] asserted that due to excessive demand for tourism destinations in the congestion model, those tourists who perceive congestion tend to choose other destinations [76]. Dissatisfaction derived from congestion leads either to visiting less-congested areas, or to becoming a “congestion avoider” who gives up on individual tourism [29]. Many such studies on coping behavior have defined behaviors of searching for and moving to less-congested destinations and travelling to completely different areas [77,78,79,80]. The coping behavior from the congestion model provides substantial implications for outdoor recreation destinations, such as national parks, which have had many visitors even in the aftermath of COVID-19. Given these implications, follow-up research is required to verify the causal relationship between risk-reducing behaviors, such as how tourists respond when they are in danger by adjusting their behaviors, such as refraining from travel or avoiding crowded places, to decrease perceived risk [72,81,82,83].

The coping behavior of visitors to national parks related to the perception of risk of COVID-19 in our study is defined as an effort to reduce factors of risk on trails. The major social-distancing and quarantine measures used to respond to COVID-19, in addition to the mandatory wearing of masks, were to reduce congestion in specific destinations or to minimize face-to-face contact according to each stage (weak stage 1 to strong stage 3), but tourists at parks can essentially make their own guidelines to cope with the risks [84,85,86]. Individuals as tourists or consumers come up with their own countermeasures to reduce risks [31,34,71,72] which can be described as “behavior for risk reduction” or “strategies for risk reduction.” Risk reduction also occurs when consumers make purchases in a way that reduces uncertainty or dissatisfaction by using strategies to minimize adverse outcomes and to abate potential risks [87].

## 3. Methods

### 3.1. Research Model and Hypotheses

**Figure 1:**
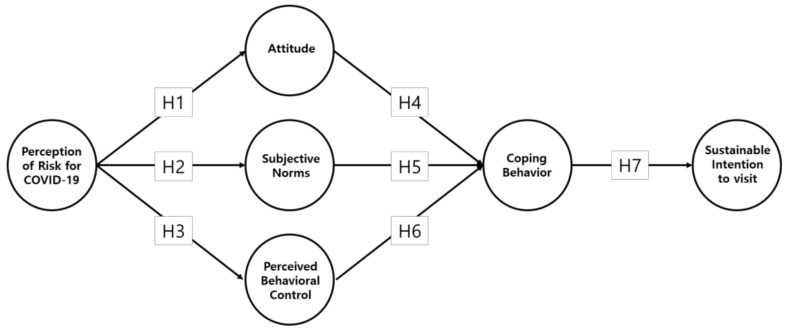
Research model.

Given the previous studies, a research model was developed to explain tourists’ sustainable intention to visit a national park, and seven hypotheses were established (Figure 1). Hypotheses 1–3 explain that perception of risk of contracting COVID-19 has a significant effect on attitude, subjective norms, and perceived behavioral control [67,69].

**Hypothesis** **1.** **(H1):** *Perception of risk from COVID-19 significantly affects attitude*.

**Hypothesis** **2.** **(H2):** *Perception of risk from COVID-19 significantly affects subjective norms*.

**Hypothesis** **3.** **(H3):** *Perception of risk from COVID-19 significantly affects perceived behavioral control*.

Hypotheses 4–6 propose that attitude, subjective norms, and perceived behavioral control significantly influence coping behavior. These were established based on extant discussion on the relationships between psychological variables such as attitude and coping behavior [10,73,74,88]. Additionally, hypothesis 7 was developed to find out whether coping behavior has a significant effect on sustainable intention to visit. Our definition of a sustainable intention to visit is a continuous intention to visit national parks.

**Hypothesis** **4.** **(H4):** *Attitude significantly affects coping behavior*.

**Hypothesis** **5.** **(H5):** *Subjective norms significantly affect coping behavior*.

**Hypothesis** **6.** **(H6):** *Perceived behavioral control significantly affects coping behavior*.

**Hypothesis** **7.** **(H7):** *Coping behavior significantly affects sustainable intention to visit*.

### 3.2. Data Collection and Analytical Methods

#### 3.2.1. Instrument

The questionnaire was composed of 36 questions, accounting for the six variables presented in the hypotheses and the demographic variables of the sample. The measurement items were derived from the literature review and were determined after discussion and revision of items with three professionals in the tourism field. Specifically, the five items for measuring perception of risk from COVID-19 were reconstructed by extracting variables for health threats [30,39,40,41]. The three items for measuring coping behavior were adapted from research on congestion avoidance [89,90]. Finally, the items about the theory of planned behavior, such as attitude, subjective norms, perceived behavioral control, and visiting intention, were adapted from other extant studies [13,22,23,42,91,92].

#### 3.2.2. Data Collection and Statistical Tools

The survey was conducted at parking lots at the entrance to Boriam, which is located in Hallyeohaesang National Park, Namhae-Gun, Korea. In 1968, Hallyeohaesang National Park was designated as the fourth national park in Korea. This park is a treasure trove of marine ecosystems in which large and small islands and natural sceneries harmonize. The total area of the park is 545.63 km^2^, and 72.3% of the total consists of sea area (https://english.knps.or.kr/, accessed on 15 July 2020). Boriam is the only mountain park in Korea located within 650 m of the Hallyeohaesang National Park and can be accessed within an hour from surrounding cities, such as Yeosu, Gwangyang, Suncheon, Sacheon, Jinju, Namhae, and Tongyeong. It is recognized as a place where visitors can enjoy their stays while complying with COVID-19 measures, because there is not a dense dining zone as is commonly found in the national park areas in Korea. The survey was conducted on 26 to 27 September 2020, targeting visitors who were over 20 years old, in cooperation with the Hallyeohaesang National Park Office. Visitors were intercepted and asked to answer the questionnaires by ten trained interviewers. The survey was self-administered with a convenient sampling method. A total of 370 questionnaires were distributed and collected. Of these, 351 samples were used for the analyses, excluding 19 unusable samples with missing values. Data analyses were performed using SPSS Statistics version 23 and AMOS 18.0. The validity of the measurement variables was tested by means of confirmatory factor analysis and correlation analysis. The reliability was tested using Cronbach’s alpha. Both testing the research hypotheses and comparing the two research models were conducted by structural equation modelling. Additionally, the parsimonious fit index (PFI) and squared multiple correlations (SMC) were calculated to identify significant improvements from the two research models.

## 4. Results

### 4.1. Demographic Information of the Sample

Frequency analysis was conducted to identify demographic characteristics, which included gender, residence, age, occupation, monthly income, and educational level, of the 351 usable subjects (Table 1). For gender, there was no significant difference between males (50.7%) and females (49.3%) in their proportions of respondents, and their ages showed an even distribution that ranged from the 20s to over 60s. The most common monthly income included 130 respondents (37.0%), who made more than 5000 USD, followed by the group making 4000–5000 USD comprising 83 respondents (23.6%). In occupation, the most common group was 83 respondents (23.6%) with employees, followed by 52 respondents (14.8%) who were professionals or housewives. Regarding the educational level of the respondents, the most common group comprised those with 4-year college degrees (80 respondents, 55.4%), followed by the group with 2-year college degrees (37 respondents, 25.2%). For place of residence, Kyungnam, adjacent to the survey location, was the most common, with 30 respondents (20.4%), followed by 13 respondents (8.8%) from Jeonnam, and 11 respondents (7.5%) from Busan.

### 4.2. Validity and Reliability of Measurements

As shown in Table 2, the confirmatory factor analysis to verify the validity of the measurement items showed that the fit index of the measurement model was χ^2^/df = 2.493 (χ^2^ = 760.377, df = 305), RMR = 0.043, RMSEA = 0.064, GFI = 0.869, NFI = 0.907, RFI = 0.893, IFI = 0.943, TLI = 0.934, and CFI = 0.943, thereby meeting the statistical criteria. In general, the recommended criteria for the fit index are presented as χ^2^/df < 3, RMSEA < 0.08, RMR < 0.05, GFI, NFI, TLI, CFI > 0.9 [93,94]. After verification of the convergent validity of the measurement items, one of the items used to measure the perception of risk from COVID-19 did not meet the criterion (0.5–0.95) and so was deleted. The deleted item was about whether “there was an atmosphere of refraining from visiting the national park because of COVID-19,” showing 0.375 of the factor loading.

As a result of re-conducting the confirmatory factor analysis after removing the questionnaire item that did not meet the criterion of factor loading, the fit index of the measurement model was χ^2^/df = 2.420 (χ^2^ = 677.548, df = 208), RMR = 0.031, RMSEA = 0.064, GFI = 0.874, NFI = 0.913, RFI = 0.901, IFI = 0.947, TLI = 0.938, and CFI = 0.947, showing that most of the fit indices improved slightly. The convergent validity was suitable for the model, because all values of composite reliability (CR), factor loading, and average variation extracted (AVE) for each measurement were higher than the criteria. Additionally, correlation analysis between measurement items, as shown in Table 3, showed that the correlation coefficients between measurement items were between 0.100 and 0.769, thereby meeting the recommended criterion of 0.85 [95]. The AVEs of each measurement item were larger than the squared values of the correlation coefficients, confirming that there was discriminant validity [96,97].

### 4.3. Testing the Hypotheses

Structural equation modeling was performed to verify research hypotheses 1–7 with the entire sample (*n* = 351) (Table 4). To begin with, by assessing the values of χ^2^/df, RMR, CFI, TLI, RMSEA, and NFI, which are commonly provided to evaluate the fit of a structural model, it was found that they all met the criteria. Testing the research hypotheses showed that the perception of risk of COVID-19 had a significant effect on both attitude and perceived behavioral control; hence, hypotheses 1 and 3 were accepted. Hypothesis 2, however, was rejected, because the perception of risk of COVID-19 did not significantly influence subjective norms. From these results, we assume that the perception of risk of COVID-19 has a negative effect on the psychological variables of individuals, such as attitudes and perceived behavioral control toward visiting national parks, eventually leading to a decrease in visitors.

Among the major variables of the theory of planned behavior, subjective norms and perceived behavioral control had significant effects on coping behavior; hence, hypotheses 5 and 6 were accepted. As the effect of perceived behavioral control is the highest estimate, the result from our study parallels those in previous studies [13,23,67,98,99,100]. As attitude did not significantly affect coping behavior, however, hypothesis 4 was rejected. What our study indicates is that hypotheses 4 and 5 explain whether attitude and subjective norms significantly affect coping behavior, as mirrored in the distinct case of the COVID-19 pandemic. In particular, what our study found by testing hypothesis 4 differs from the results in a few other studies in which the theory of planned behavior was applied. That is, if an individual belongs to a particular group, the dynamics and norms within the group allow less freedom in the individual’s attitudes and behaviors [100,101,102]. The concern that an infectious disease might spread to the affiliated group functioned as a norm, thereby weakening individual free will—for instance, via attitude.

Mayo and Jarvis (1982) [103] also argued that the influence of an individual’s attitude may be unstable, depending on particular circumstances, and that the opinion of the affiliated group can influence individual behavior in order to be faithful to the role assigned to society. The interaction between the subjective norms and coping behavior proposed in hypothesis 5 can be accounted for in a similar context. SARS-CoV-2 has primarily infected groups with close social relationships, such as family, friends, and work. Decision making by the affiliated group, therefore, plays an important role in participating in particular tourism activities. The result of testing hypothesis 6 was an optimistic bias that individuals are relatively safe from risks that may occur in tourism destinations [41,104]. In the field of health and environment, there is literature showing that the level of perception that risks can be controlled by individuals is more common than in the field of safety accidents [30,105]. Last, because coping behavior had a significant effect on sustainable intention to visit the national park, hypothesis 7 was accepted.

### 4.4. Testing the Research Model Fit

The research model (Model B) for our study was compared with the base model (Model A) by adding coping behavior as a variable to the theory of planned behavior during the COVID-19 pandemic. Parsimonious fit index (PFI) and squared multiple correlations (SMC) were derived to verify and compare the two research models. When the two models were compared after a variable of coping behavior was added to the research model in our study, one index of the PFIs was found to be better, and the SMC was significantly improved (see Table 5). The PFI can be used as a criterion for judging which of two or more research models is more suitable in a case where the research model is complicated due to the addition of variables [106,107]. The PFIs such as parsimonious goodness of fit index (PGFI), parsimonious normed fit index (PNFI), parsimonious comparative fit index (PCFI), and Akaike information criterion (AIC) are provided as criteria for determining the research model’s fit. Note that in each of those cases, the lower the value, the better the research model [108]. Additionally, the SMC refers to the proportion of the total variation explained by the model [109]. Resultantly, the research model of our study (Model B) had higher PGFI and AIC than the basic research model (Model A), though two models presented the same PCFI. For our research model, the PNFI was lower, and the SMC that explains sustainable intention to visit was 75.6%, which was higher than that of the extant research model (49.4%). The research model, therefore, that accounts for sustainable intention to visit the national park under the COVID-19 pandemic, achieved a similar PFI value to that of the basic research model, despite the addition of a variable which is coping behavior. Additionally, the suitability of the Model B was verified because the SMC was improved.

## 5. Conclusions

Our study originated in a research question about why visitors continue to visit natural destinations such as national parks during the COVID-19 pandemic. In addition, the purpose of our study was to analyze an extended research model of whether visitors intend to continue to visit a certain national park by minimizing individual risks by means of coping behavior, such as walking on less-congested trails and minimizing face-to-face contact. A research model was developed to explain the relationships among perception of risk, variables from the theory of planned behavior, and coping behavior based on previous studies [10]. After seven hypotheses originating from the research model were established, they were tested. Five were accepted. Perception of risk of COVID-19 had a significant effect on attitude and perceived behavioral control. Subjective norms and perceived behavioral control had significant effects on coping behavior. Finally, there was a significant relationship between coping behavior and sustainable intention to visit. The conclusions and theoretical implications of our study are as follows.

First, in both models, the perception of risk of COVID-19 had a negative effect on both attitude and perceived behavioral control, which are psychological variables that either directly or indirectly induce behaviors. This implies that the results are linked to reasons for the decrease in visitors to many tourism destinations, including national parks, during the worldwide pandemic. The perception of risk of COVID-19 did not significantly affect the subjective norms in either model, which indicates that, because subjective norms are the thoughts by those members of particular groups, the perception of risk of COVID-19 is not an independent variable that can explain the subjective norms. Second, in Model A, attitude did not have a significant effect on sustainable intention to visit, whereas the subjective norms did have a significant effect on sustainable intention to visit. These results imply that the influential factor that sustains an intention to visit during the COVID-19 pandemic is an evaluation of the given surroundings, such as by subjective norms, not the temporarily impotent attitude. The social attitude that outdoor natural tourism destinations such as national parks are safer than indoor counterparts is delivered to the affiliated group, thereby making tourism activities available.

Third, perceived behavioral control in Model A had the greatest influence on sustainable intention to visit, apparently because visitors with perceived behavioral control decide to visit safer national parks rather than other destinations, even during the COVID-19 pandemic. Boriam in Hallyeohaesang National Park, which was the location of data collection, is a destination that residents in a few cities near Boriam can easily visit in daily life with their vehicles. It consists of trails that facilitate self-control measures, such as by social distancing, due to their relatively low congestion, which indicates that the perceived behavioral control of knowing about trails is a major factor in visiting national parks.

Fourth, testing the fit of Model B showed that adding explanatory variables to the variables already included in the extant theory should be continued for human planned behavior. The expansion of the theory of planned behavior applied in our study began from a criticism of the low explanatory power of three variables: attitude, subjective norms, and perceived behavioral control [54,57]. Despite the addition of two variables—perception of risk and coping behavior for COVID-19—the PFI of Model B reached a favorable level, and an improvement in explanatory power was a result consistent with the original purpose of expanding the theory of planned behavior. In particular, although the variable of coping behavior in Model B helped improve the explanatory power, the question of whether coping behavior fully plays a mediating role between the effects of the original predictors and the intentions remains. Follow-up studies are necessary to identify direct and indirect effects of predictors on the intentions in the proposed models. Furthermore, because both the results showed good fit indices from Models A and B, it indicates that both models are available to apply to a special situation as the COVID-19 pandemic. Other studies also tell us the usefulness of the extended theory of planned behavior [110].

In addition, the result from our study that subjective norms and perceived behavioral control have significant effects on coping behavior provides worthwhile clues that explain the psychological changes they drive when visitors come to national parks during the pandemic in the East. The statistics on the number of visitors to national parks in Korea did not show a large decline in visitors compared to other tourism destinations in 2020. This implication needs to be analyzed in more detail in future studies, because it might arise from reasons for choosing natural tourism destinations such as national parks being recognized as relatively safe during the COVID-19 pandemic, having infrastructure that has matured, such as mountaineering and trekking facilities, having online information available, and encouraging those who comply with quarantine rules to visit tourism destinations.

The results from our study provide not only theoretical, but practical implications for the efficient control and management of visitors to national parks in response to the COVID-19 pandemic. First, national parks enable visitors to visit because of the optimistic idea that national parks are safer than are other tourism destinations, and because individuals may know a lot from prior information collection about destinations. Rather than the consistent social distancing mandated by the government based on increases in the number of infected, therefore, what is needed is management of visitors by means of assertive alternatives, such as entry and exit control by time. Second, a natural resource such as a national park can become a means to alleviate the side effects that result in Corona Blues caused by the pandemic [111]. If the quarantine stage of social distancing is eased because of a decrease in the number of the infected, a plan to distribute the number of visitors, for example, by temporarily opening some of the national park trails operated as a nature rest-year system, should also be considered while meeting the needs of visitors. Finally, in order to provide convenience for potential visitors, it is essential to develop a smart application related to congestion management that can support the congestion indexing of individual trails in national parks in real time.

Our study, being empirical, has the following limitations. First, the results cannot be generalized, due to the temporal and spatial limitations. In order to improve the reliability and validity of the results from our study and to generalize them, a longitudinal study should be conducted, not only in Hallyeohaesang National Park where the data collection of our study was conducted in 2020, but also in other national parks in Korea. Additionally, future research that compares the impacts of the COVID-19 pandemic on tourism area before and during COVID-19 will be meaningful [112]. Second, a few particular variables in the research model of our study did not show seamless causal relationships. As recent studies on the COVID-19 pandemic have proliferated in the tourism field and have accumulated, future research to generalize the results from our study should be carried out. Third, questions measuring sustainable intention to visit were not adequate in that a typical question for such an item should be, “I intend to visit the national park.” The items such as “I will visit the national park” that have been used in our study could produce a violation of the compatibility of the principles of the theory of planned behavior. These items, therefore, should be revised for adequate measurements in future research. Lastly, no significant effect was found in the relationship between attitude and coping behavior in the research model: *p* = 0.057, which is close to the 5% significance level. It is imperative to further verify whether the results from our study are similar to those in other cases.

## Figures and Tables

**Table 1:** Demographic characteristics of the sample.

Item		*n* (%)	Item		*n* (%)
Gender	Male	178 (50.7)	Educational level	Under high school	16 (10.2)
	Female	173 (49.3)		2-year college	37 (25.2)
Age	20s	75 (21.4)		4-year college	80 (55.4)
	30s	54 (15.4)		graduate	14 (9.5)
	40s	71 (20.2)	Residence	Seoul	8 (5.4)
	50s	91 (25.9)		Busan	11 (7.5)
	More than 60s	60 (17.1)		Daegu	12 (8.2)
Monthly income	Less than 1000 USD	4 (1.1)		Incheon	5 (3.4)
	1000–2000 USD	13 (3.7)		Gwangju	7 (4.8)
	2000–3000 USD	54 (15.4)		Daejeon	8 (5.4)
	3000–4000 USD	67 (19.1)		Ulsan	6 (4.1)
	4000–5000 USD	83 (23.6)		Kyounggi	12 (8.2)
	More than 5000 USD	130 (37.0)		Kangwon	3 (2.0)
Occupation	Self-employer	41 (11.7)		Chungbuk	4 (2.7)
	Professional	52 (14.8)		Chungnam	8 (5.4)
	Official	44 (12.5)		Jeonbuk	11 (7.5)
	Farmer/fisherman	14 (4.0)		Jeonnam	13 (8.8)
	Student	33 (9.4)		Kyungbuk	5 (3.4)
	Housewife	52 (14.8)		Kyungnam	30 (20.4)
	Employee	83 (23.6)		Jeju	1 (0.7)
	Others	32 (9.1)		Sejong	3 (2.0)

**Table 2:** Convergent validity and reliability of the measurements.

Factor	Items	S.E.	*t*	Standardized Coefficients	AVE	CR	Cronbach’s α
Perception of Risk for COVID-19	Trails of the national park are not safe from COVID-19	0.080	11.705 ***	0.652	0.609	0.861	0.812
	There is insufficient information on visiting the national park under COVID-19	0.074	13.479 ***	0.747			
	There are concerns about the quarantine and hygiene conditions of indoor facilities such as restrooms and shelters in the national park	0.084	14.743 ***	0.839			
	There are not enough tour programs where visitors can safely participate in under COVID-19	-	-	0.771			
Attitude	I like to visit the national park	0.050	20.404 ***	0.869	0.865	0.970	0.950
	I am happy to visit the national park	0.040	23.797 ***	0.869			
	I am positive about visiting the national park	0.037	25.453 ***	0.893			
	Visiting the national park is worthwhile	0.037	27.460 ***	0.922			
	Visiting the national park will give me good outcomes.	-	-	0.896			
Subjective Norm	My family thinks positively about my visit to the national park	0.045	18.073 ***	0.768	0.837	0.968	0.950
	My friends think positively about my visit to the national park	0.041	23.588 ***	0.880			
	My acquaintances think positively about my visit to the national park	0.040	25.202 ***	0.907			
	My family will want me to visit the national park	0.042	23.257 ***	0.874			
	My friends will want me to visit the national park	-	-	0.885			
	My acquaintances will want me to visit the national park	0.030	32.685 ***	0.870			
Perceived Behavioral Control	I can visit the national park whenever I want	0.088	13.907 ***	0.798	0.636	0.897	0.865
	I am financially able to afford to visit the national park	0.077	13.735 ***	0.787			
	I have enough time to visit the national park	0.088	13.460 ***	0.770			
	It is easy to learn necessary skills for visiting the national park	0.086	11.929 ***	0.680			
	I can easily find the information about visiting the national park	-	-	0.717			
Sustainable Intention to Visit	I will try to continue to visit the national park	0.039	25.448 ***	0.927	0.887	0.959	0.921
	I will recommend visiting the national park to others	0.036	22.871 ***	0.873			
	I am sure I will continue to visit the national park	-	-	0.885			
Coping Behavior	I will choose trails that are expected to have fewer visitors.	0.102	12.585 ***	0.717	0.678	0.863	0.786
	I will minimize to spend time where other visitors gather on trails	0.113	11.687 ***	0.849			
	When visiting, I will try to comply with the rules on COVID-19	-	-	0.708			

*** *p* < 0.000.

**Table 3:** Correlations between measurements.

Category	Perception of Risk for COVID-19	Attitude	Subjective Norms	Perceived Behavioral Control	Coping Behavior	Sustainable Intention to Visit	AVE
Perception of Risk for COVID-19	1						0.609
Attitude	−0.201 (0.040)	1					0.865
Subjective Norms	−0.100 (0.010)	0.769 (0.591)	1				0.837
Perceived Behavioral Control	−0.236 (0.056)	0.667 (0.445)	0.623 (0.388)	1			0.636
Coping behavior	0.122 (0.015)	0.390 (0.152)	0.402 (0.162)	0.371 (0.138)	1		0.678
Sustainable Intention to visit	−0.114 (0.013)	0.604 (0.365)	0.620 (0.384)	0.637 (0.406)	0.567 (0.321)	1	0.887

Note: () denotes the square of the correlation coefficient.

**Table 4:** Results of testing hypotheses by means of SEM.

Path		Estimate	S.E.	*t*	*p*
H1	Perception of Risk for COVID-19 → Attitude	−0.202	0.061	−3.410 ***	0.000
H2	Perception of Risk for COVID-19 → Subjective Norm	−0.101	0.061	−1716	0.086
H3	Perception of Risk for COVID-19 → Perceived Behavioral Control	−0.233	0.055	−3.755 ***	0.000
H4	Attitude → Coping Behavior	0.187	0.059	2.184 *	0.029
H5	Subjective Norm → Coping Behavior	0.304	0.057	3.685 ***	0.000
H6	Perceived Behavioral Control → Coping Behavior	0.344	0.062	4.517 ***	0.000
H7	Coping Behavior → Sustainable Intention to Visit	0.870	0.113	11.604 ***	0.000

* *p* < 0.05, *** *p* < 0.001.

**Table 5:** Results of testing the research model’s fit.

Model		Parsimonious Fit Index				Squared Multiple Correlations (SMC)
		PGFI	PNFI	PCFI	AIC	
A	Perception of Risk for COVID-19 + Theory of Planned Behavior	0.694	0.792	0.816	643.596	0.494
	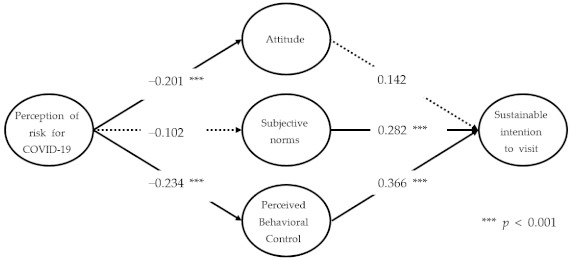					
	Goodness of fit for the model: χ^2^/df = 2.424, RMR = 0.027, CFI = 0.956, TLI = 0.948, RMSEA = 0.064, NFI = 0.928					
B	Perception of Risk for COVID-19 + Theory of Planned Behavior + Coping Behavior	0.698	0.787	0.816	797.152	0.756
	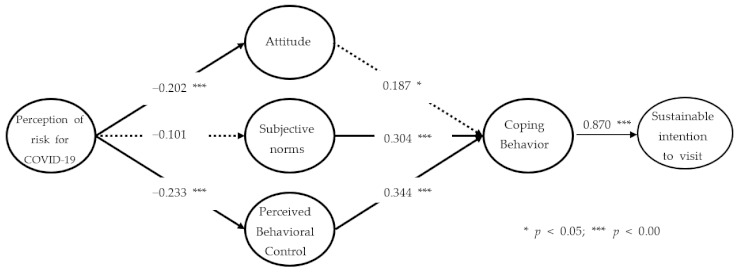					
	Goodness of fit for the model: χ^2^/df = 2.341, RMR = 0.037, CFI = 0.950, TLI = 0.942, RMSEA = 0.062, NFI = 0.916					

Note: A denotes the prior research model, and B denotes the research model in our study.

## Data Availability

The datasets used in this research are available upon request from the corresponding author. The data are not publicly available.
